# Supplementary material for: Initiation of cytosolic plant purine nucleotide catabolism involves a monospecific xanthosine monophosphate phosphatase
Source: Nat Commun. 2021 Nov 25;12:6846. doi: 10.1038/s41467-021-27152-4 (PMC8616923; doi:10.1038/s41467-021-27152-4)
Supplement: Supplementary file 1 — Supplementary Information [file 41467_2021_27152_MOESM1_ESM.pdf]

Initiation of cytosolic Plant Purine Nucleotide Catabolism involves a monospecific Xanthosine Monophosphate Phosphatase

Katharina J. Heinemann, Sun-Young Yang, Henryk Straube, Nieves Medina-Escobar, Marina Varbanova-Herde, Marco Herde, Sangkee Rhee, Claus-Peter Witte

SUPPLEMENTARY INFORMATION

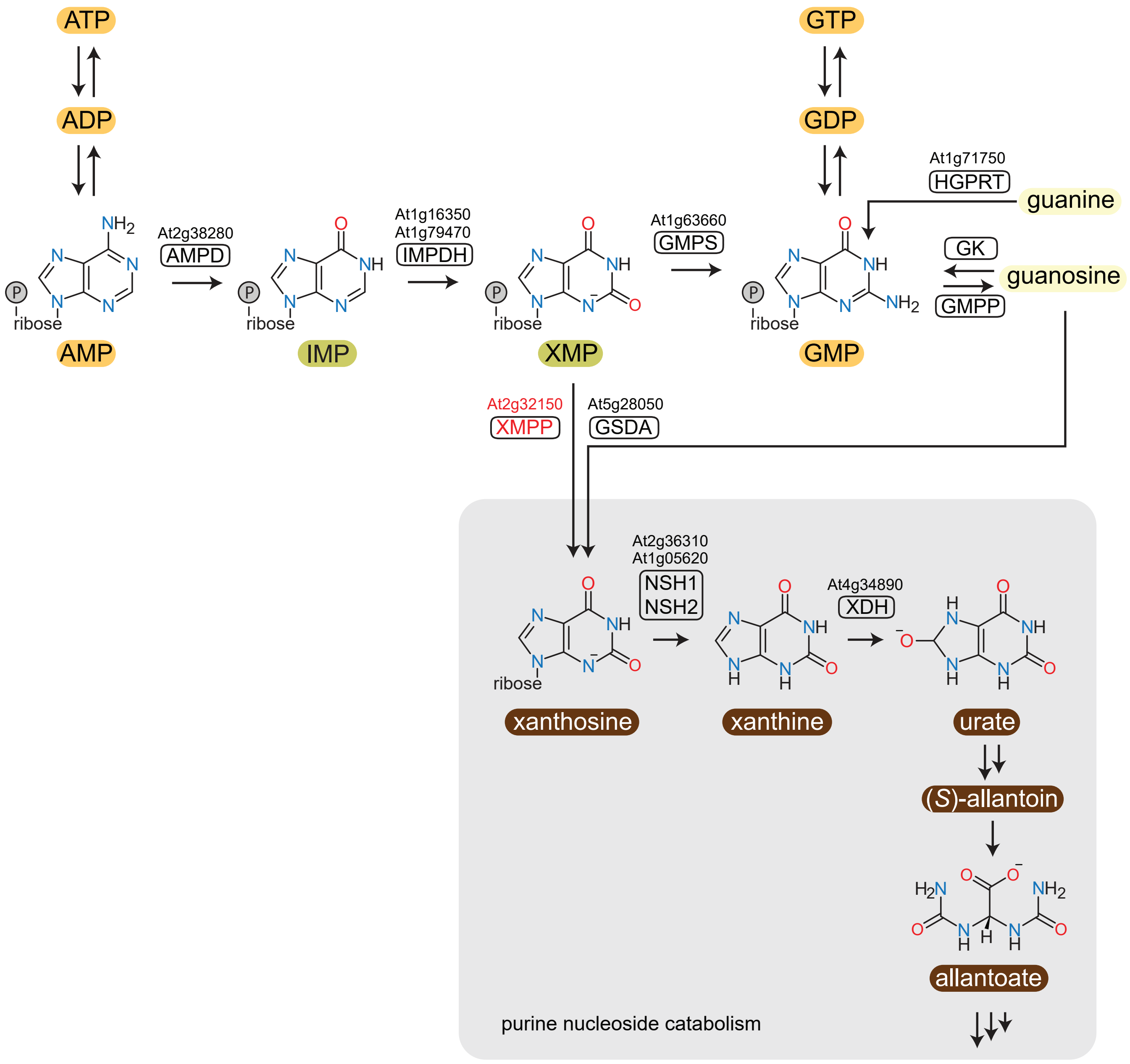

Supplementary Fig. 1. Extended scheme of GMP biosynthesis and purine nucleotide catabolism. Labels are as in Fig. 1a. The possible interconversions of the adenosine and guanosine triphosphates, diphosphates and monophosphates are additionally shown. Bases are drawn in their ionic form when they are mainly present as ions at neutral pH.

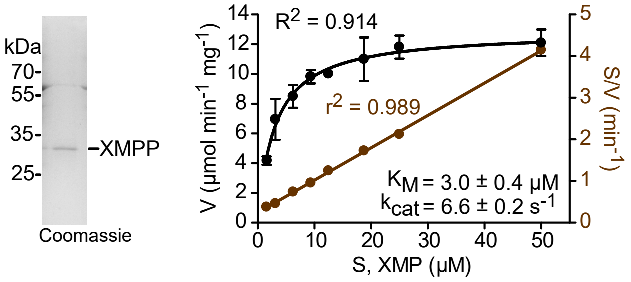

Supplementary Fig. 2. Purity and determination of kinetic constants of N-terminal tagged XMPP. Left panel, Coomassie-stained SDS-gel with the N-terminal Strep-tagged XMPP purified from leaf extracts of *Nicotiana benthamiana* after transient expression. Right panel, determination of the kinetic constants with the data fitted according to Michaelis Menten (left axis) or Hanes (right axis). Error bars are SD, n = 3 independent measurements using the same enzyme preparation.

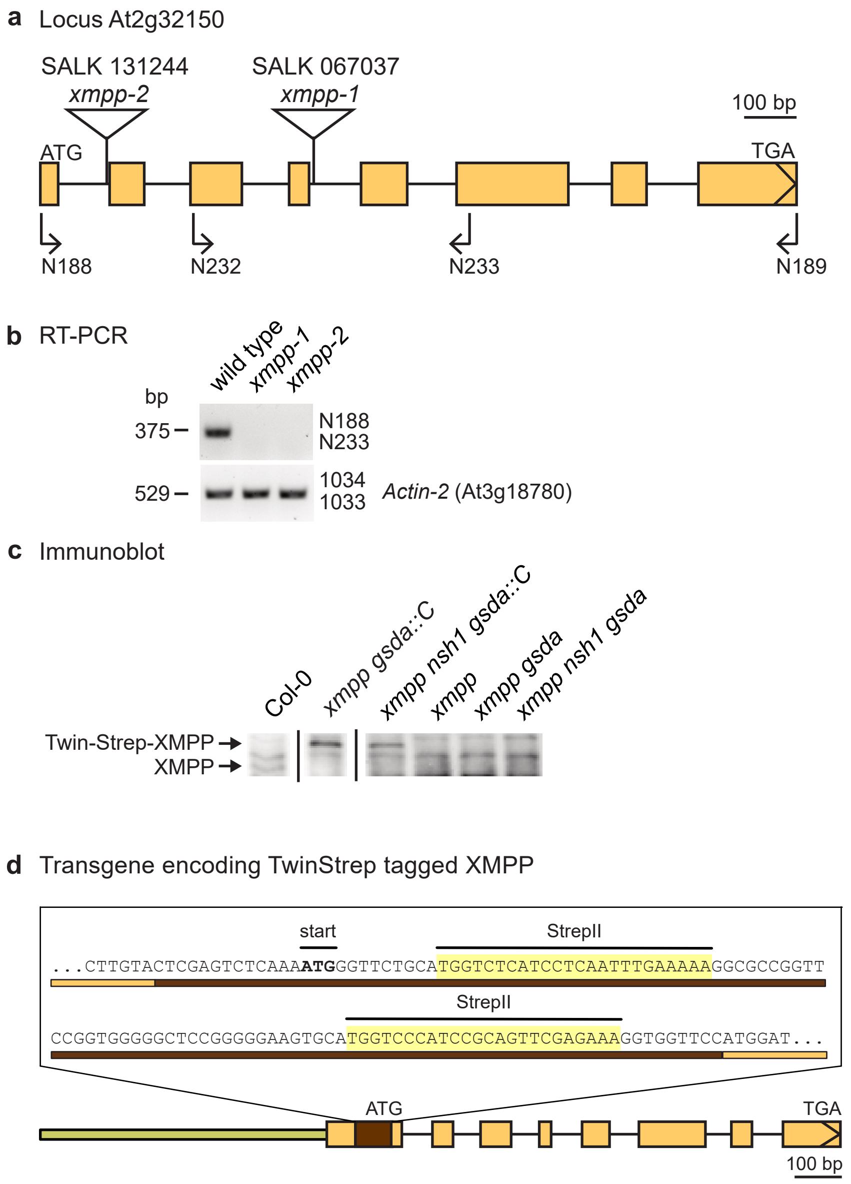

Supplementary Fig. 3. Characterization of plant lines with T-DNA insertions in *XMPP* and lines that express an *XMPP* transgene. a Coding region of the XMPP gene with exons shown as boxes and T-DNA insertion positions marked by triangles. Primers are indicated by arrows. **b** Semi-quantitative RT-PCR data showing the absence of intact *XMPP* mRNA in the two T-DNA insertion lines (representative result out of two repeats). The primers used for amplification are indicated at the right of the panels. The *actin2* mRNA (encoded at locus At3g18780) was used as positive control. **c** Immunoblot of seedling extracts from lines that express *XMPP* transgenes and from control lines (representative result out of two repeats). The blot was probed with a custom-made anti-XMPP antibody. The complementation lines contain a transgene (::C) encoding an N-terminal TwinStrep-tagged XMPP expressed by the native promoter. The positions of native untagged XMPP (in the wild type) and of tagged XMPP are indicated. **d** Structure of the *XMPP* transgene (exons are shown as orange boxes) with the inserted sequence including the coding sequence for the N-terminal TwinStrep tag (brown box or bar). The promoter region is shown in green.

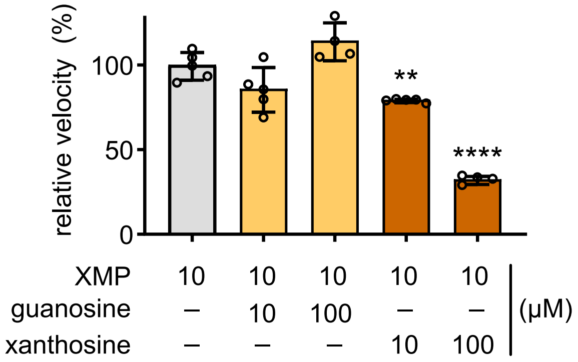

Supplementary Fig. 4. Inhibitory effect of guanosine or xanthosine on XMPP activity. The enzymatic velocity of XMPP was assessed with 10 µM XMP without any nucleosides (set to 100% at an activity of 14.0 ± 1.0 µmol mg^{-1} min^{-1}) or in the presence of 10 or 100 µM guanosine or xanthosine, respectively. Error bars are SD, n = 5 but for samples with 100 µM nucleosides n = 4 repeated assays using the same enzyme preparation. For statistical treatment, two-sided Dunnett's comparisons using the sandwich variance estimator were performed. The assay without nucleoside additions served as reference group. **, $p < 0.01$; ****, $p < 0.0001$.

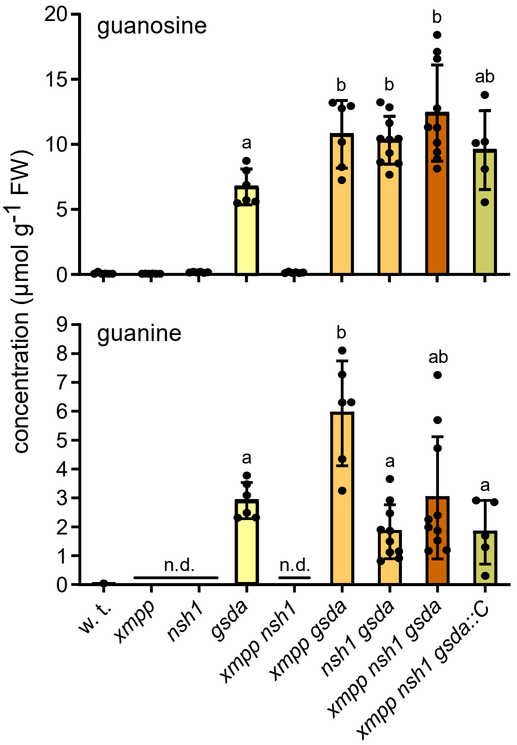

Supplementary Fig. 5. Guanosine and guanine pool sizes in *XMPP* genetic variants in the context of other mutants of purine catabolism genes in seeds. Quantification of guanosine and guanine in seed extracts of the same seeds as used in the experiment shown in Fig. 2d. The complementation line (*xmpp nsh1 gsda::C*) contains a transgene encoding an N-terminal TwinStrep-tagged XMPP expressed from a native promoter (Supplementary Fig. 3c,d). Error bars are SD; n = 6 but for *xmpp nsh1 gsda::C* n = 5 and for *nsh1 gsda* and *xmpp nsh1 gsda* n = 10. Seed samples from independent mother plants grown in parallel. Statistical analysis as in Fig. 2d.

Guanosine and guanine hyperaccumulate in *xmpp gsda* background, because additional guanosine is produced via GMP from XMP whose degradation route is blocked. Although guanine is not an intermediate of purine nucleotide catabolism in *Arabidopsis*[5], it is generated from guanosine, in part by the NSH1/NSH2 complex[2], when this metabolite accumulates in *gsda* background. A hyperaccumulation of guanosine (but not guanine) is observed also in *nsh1 gsda* seeds[2], because guanosine is less efficiently hydrolyzed to guanine and ribose due to the lack of NSH1 and because in this double mutant xanthosine accumulates (Fig. 2d) which blocks XMPP activity biochemically (Supplementary Fig. 4).

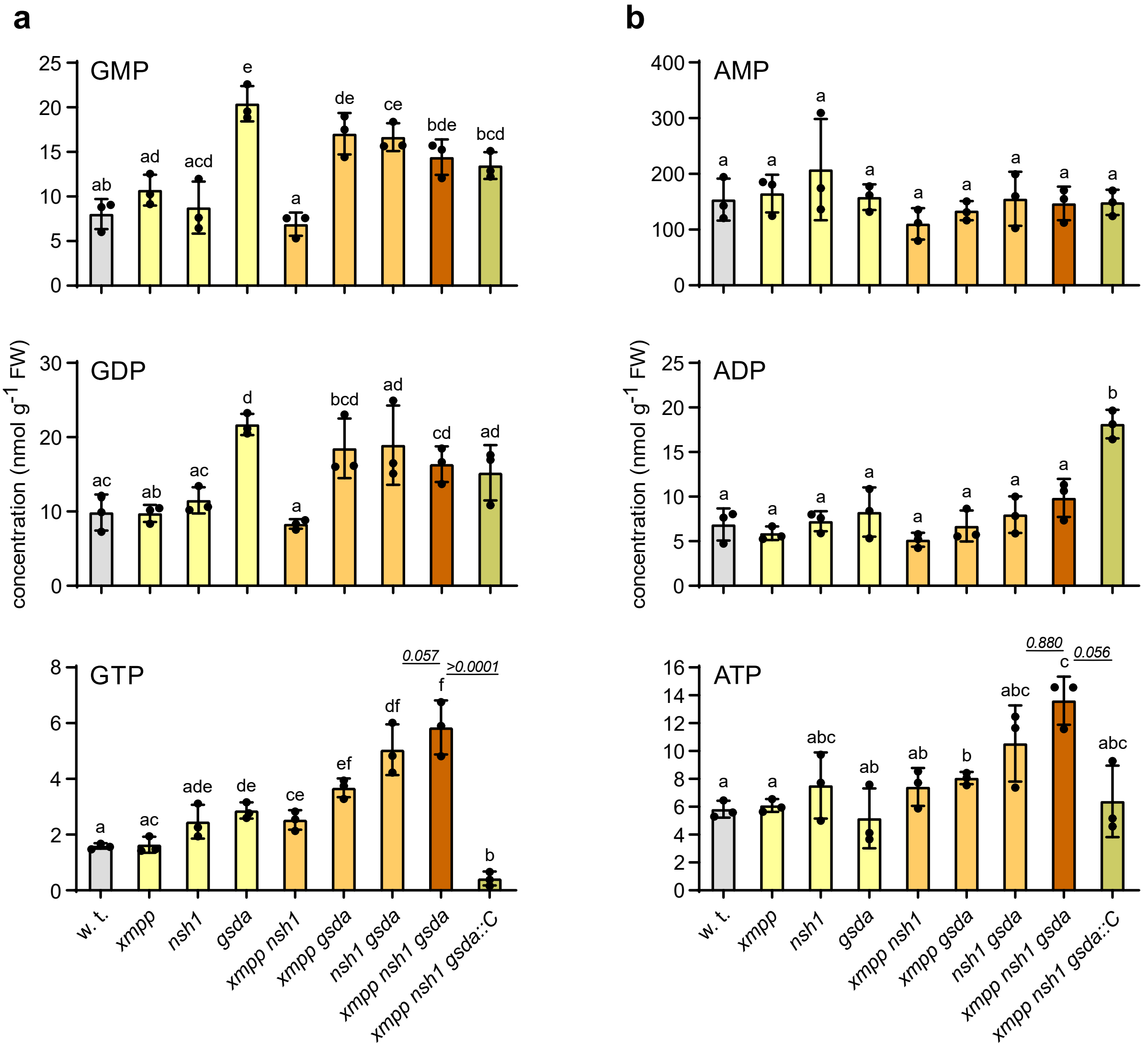

Supplementary Fig. 6. Nucleotide pool sizes in *XMPP* genetic variants in the context of other mutants of purine catabolism genes in seeds. Quantification of purine nucleotides in seed extracts of the same seeds as used in the experiment shown in Fig. 2d. **a**, GMP, GDP, GTP; **b**, AMP, ADP, ATP. Error bars are SD; n = 3 seed samples from independent mother plants grown in parallel. Statistical analysis as in Fig. 2d. Some p values are indicated above the columns in italic numbers, all p values can be found in the Source Data file.

GTP concentrations in *xmpp nsh1 gsda* seeds are even higher than in *xmpp gsda* seeds probably because guanosine hydrolysis to guanine and ribose is partially blocked in *nsh1* background (see Supplementary Fig. 5). Since guanosine degradation is compromised, more GMP is channeled into GTP.
In *nsh1 gsda* seeds, GTP concentrations are also very high, because also here guanosine hydrolysis is partially compromised. Additionally, the high xanthosine accumulation in *nsh1* (Fig. 2d) blocks XMPP biochemically (Supplementary Fig. 4) resulting in a partial molecular mimic of the *xmpp nsh1 gsda* background.

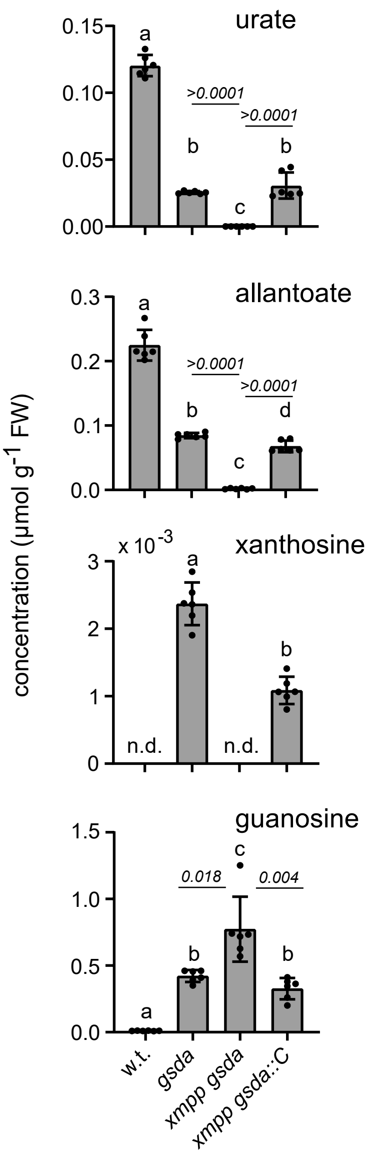

Supplementary Fig. 7. Characterization of metabolic alterations in XMPP genetic variants in the context of other mutants of purine catabolism genes in seedlings under long-day or prolonged-night conditions. This dataset includes the *xmpp gsda::C* line and is independent from similar data shown in Fig. 3. The seedlings were grown in a constant 16 hour day / 8 hour night regime up to day 5 after germination. At the end of the night of day 5, they were exposed to darkness for 48 hours. All genotypes were grown on the same plate for one biological replicate (n). Error bars are SD, n = 6.

In the complementation line *xmpp gsda::C*, urate, allantoate and guanosine pools are of similar size as in the *gsda* line, showing that the complementation by the *XMPP* transgene is effective. Comparatively small amounts of xanthosine accumulated in *gsda* background probably because the NSH1 /NSH2 nucleoside hydrolase complex is partially inhibited by the strong accumulation of guanosine in that line. This xanthosine accumulation is not observed in *xmpp gsda*, because the additional mutation of *XMPP* prevents any influx into the xanthosine pool. In the *xmpp gsda::C* complementation line, xanthosine accumulation is observed again, although the pool size is smaller than in *gsda*. The reason for this difference is unclear and might lie in the slightly different expression level of the *XMPP* transgene compared to the native *XMPP* gene (Supplementary Fig. 3c).

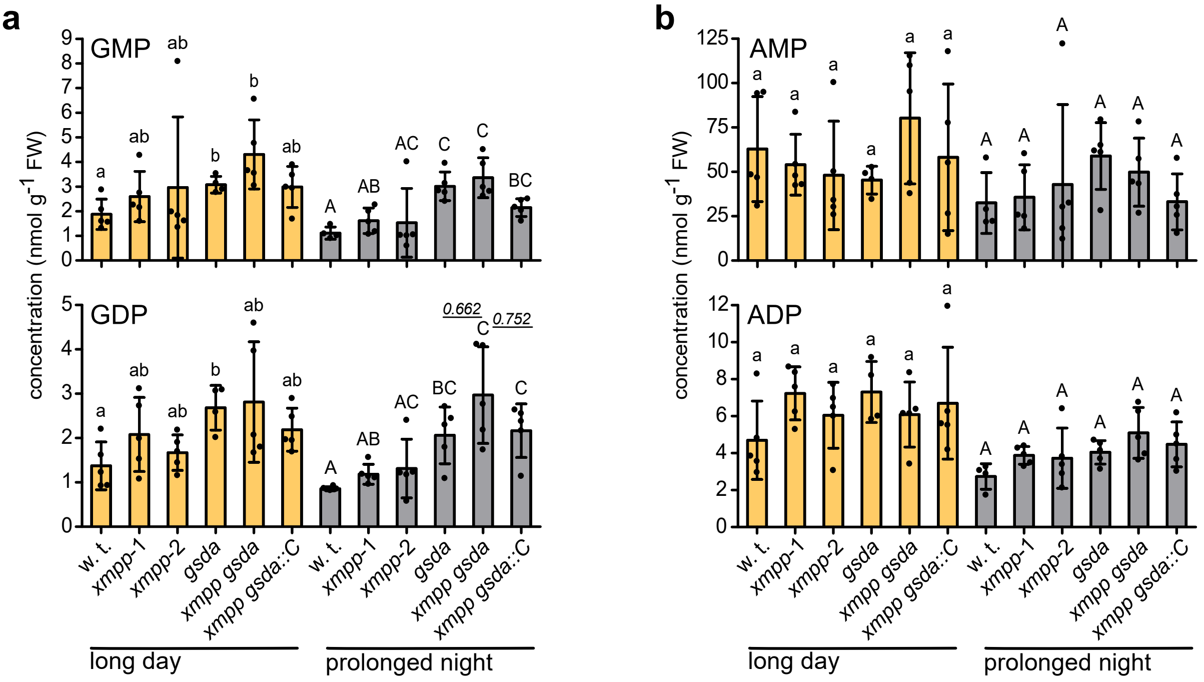

Supplementary Fig. 8. Nucleotide pool sizes in XMPP genetic variants in the context of other mutants of purine catabolism genes in seedlings under long-day or prolonged-night conditions. a GMP, GDP concentrations and **b** AMP, ADP concentrations in seedling extracts from the experiment shown in Fig. 3b. Error bars are SD, n = 5 but for *gsda* in the light and the wild type in the dark n = 4. Statistical analysis as in Fig. 3b.

The in tendency higher GTP concentration in *xmpp gsda* background (Fig. 3b) is weakly reflected at the level of GMP or GDP but not observed for AMP or ADP. Guanylates and also adenylates mostly accumulate as GTP and ATP and not in lower phosphorylation states.

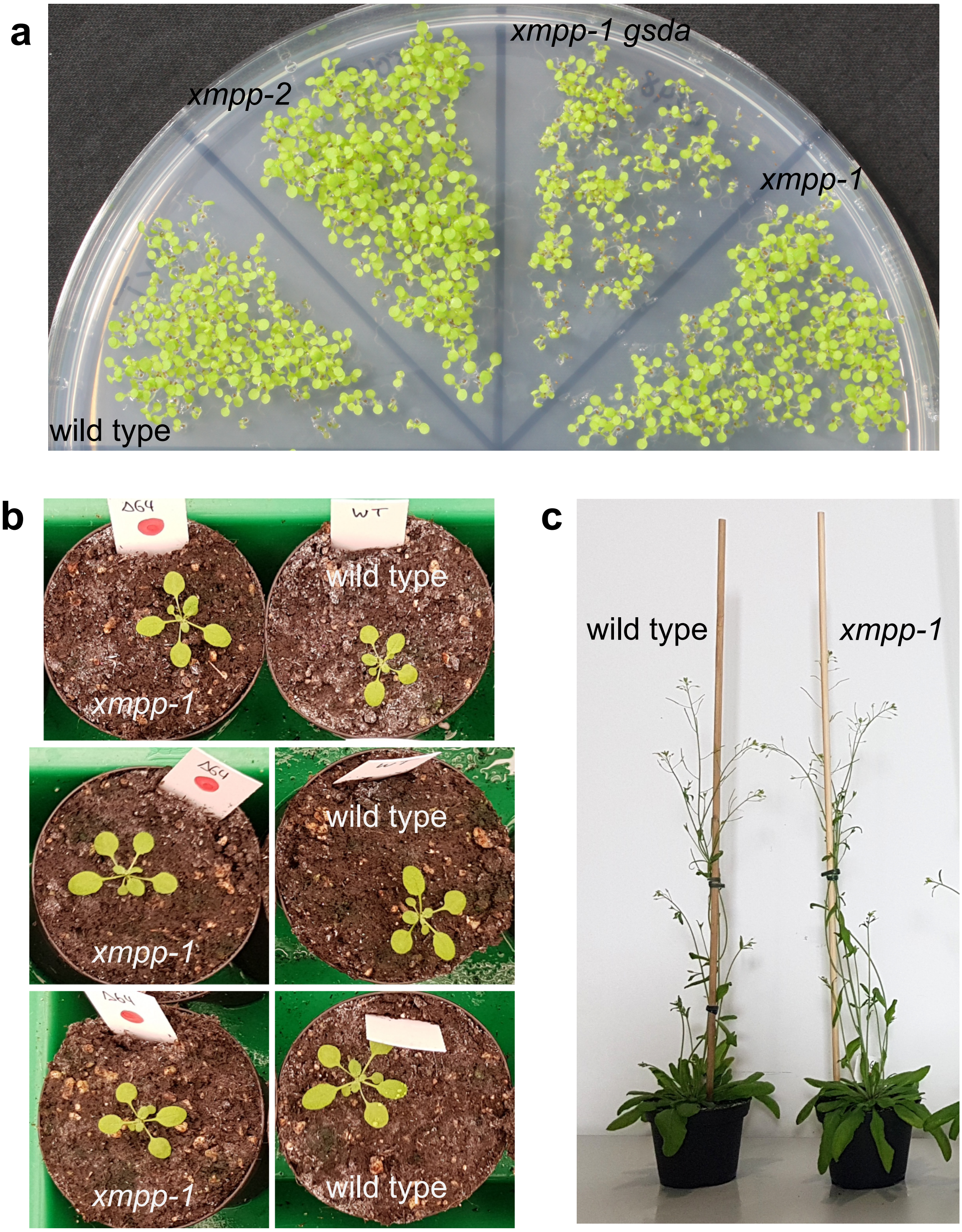

Supplementary Fig. 9. Growth of *XMPP* mutants in comparison to the wild type. All plants were grown under long day conditions with a 16 h light period. **a** 5-day-old seedlings on a half-strength Murashige and Skoog medium without sucrose and vitamins. The mutation of *GSDA* (here in *xmpp* background) is known to lead to a delay in germination[15]. **b** 17-day-old plants grown in a growth chamber. Six trays of 28 pots each containing plants with different genotypes were grown in a randomized fashion to obtain a homogenous seed batch. Wild-type and *xmpp-1* plants from one of those trays are shown. The whole tray is shown in the Source Data file. **c** Representative photograph of a wild type next to a *xmpp-1* plant in the flowering stage (40 days after germination).

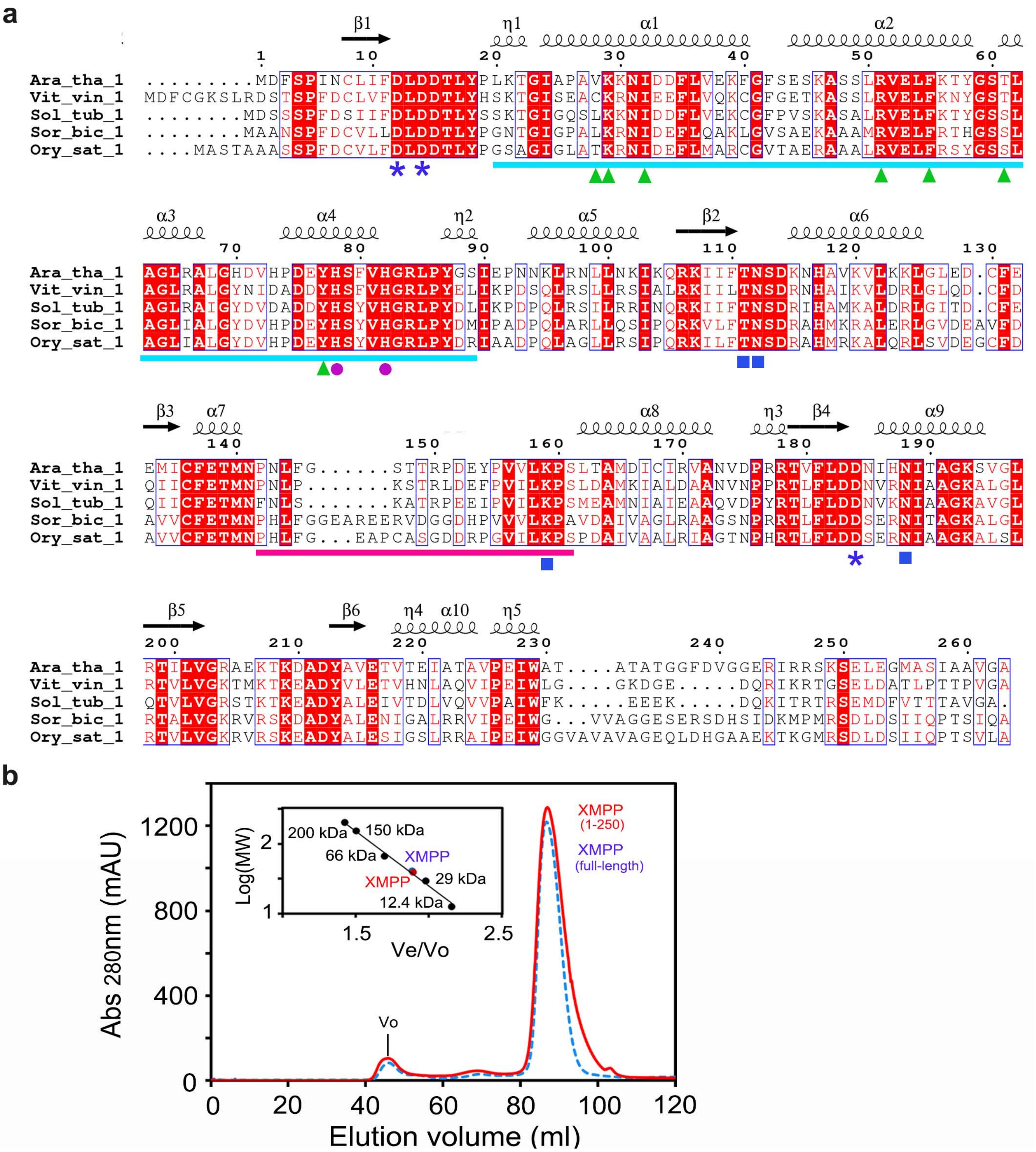

Supplementary Fig. 10. Sequence alignment and size exclusion chromatography of XMPP. **a** The amino acid sequence of XMPP from Arabidopsis (Ara_tha_1) aligned to the putative orthologs from grape (*Vitis vinifera*, Vit_vin_1), potato (*Solanum tuberosum*, Sol_tub_1), great millet (*Sorghum bicolor*, Sor_bic_1) and rice (*Oryza sativa*, Ory_sat_1). These sequences are also part of the analysis shown in Fig. 1b and are present in the XMPP/SDTL1 clade. Highly conserved residues are shown in red and boxed in blue, while strictly conserved residues are highlighted with a red background. Secondary structural elements defined in the unliganded XMPP are shown above the alignment. The cap domain and the protruding loop shown in Fig. 4a are indicated by a cyan and a red bar, respectively. Metal-binding residues are indicated by blue asterisks. Residues interacting with the phosphate, ribose and xanthine moieties of XMP are indicated by blue squares, magenta circles, and green triangles, respectively. This figure was prepared using the ESPript software[36] **b** Elution profiles from size exclusion chromatography of full-length XMPP (dashed blue line, calculated molecular weight of 29 kDa) and of the C-terminally truncated XMPP used for crystallization (solid red line, residues 1 to 250). The proteins were resolved on a Superdex 200 column (GE Healthcare) equilibrated in 50 mM Tris (pH 8.0), 100 mM NaCl, and 5% (v/v) glycerol. The void volume (V_o) was about 45 ml and the XMPP peaks corresponded to a size of ~33 kDa when calibrated with molecular weight standards of 12 to 200 kDa (insert).

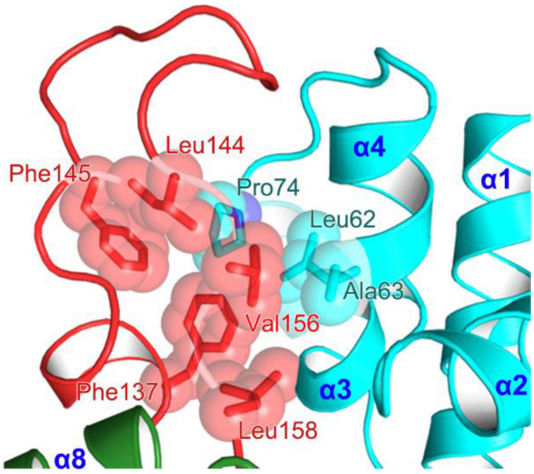

Supplementary Fig. 11. Interaction of the cap and protruding loop domains of XMPP. Hydrophobic residues with their surfaces are displayed in the unliganded XMPP. These are clustered at the interface between the cap domain and the protruding loop. The protruding loop (red) from the β3-α8 unit in the central core domain mediates extensive interactions with α3 and the N-terminal region of α4 (cyan) in the cap domain. Hydrophobic contacts of residues within 4.0 Å dominate the cap-loop interaction. Note, that the interacting hydrophobic residues are highly conserved in the XMPP homologs (Supplementary Fig. 10a).

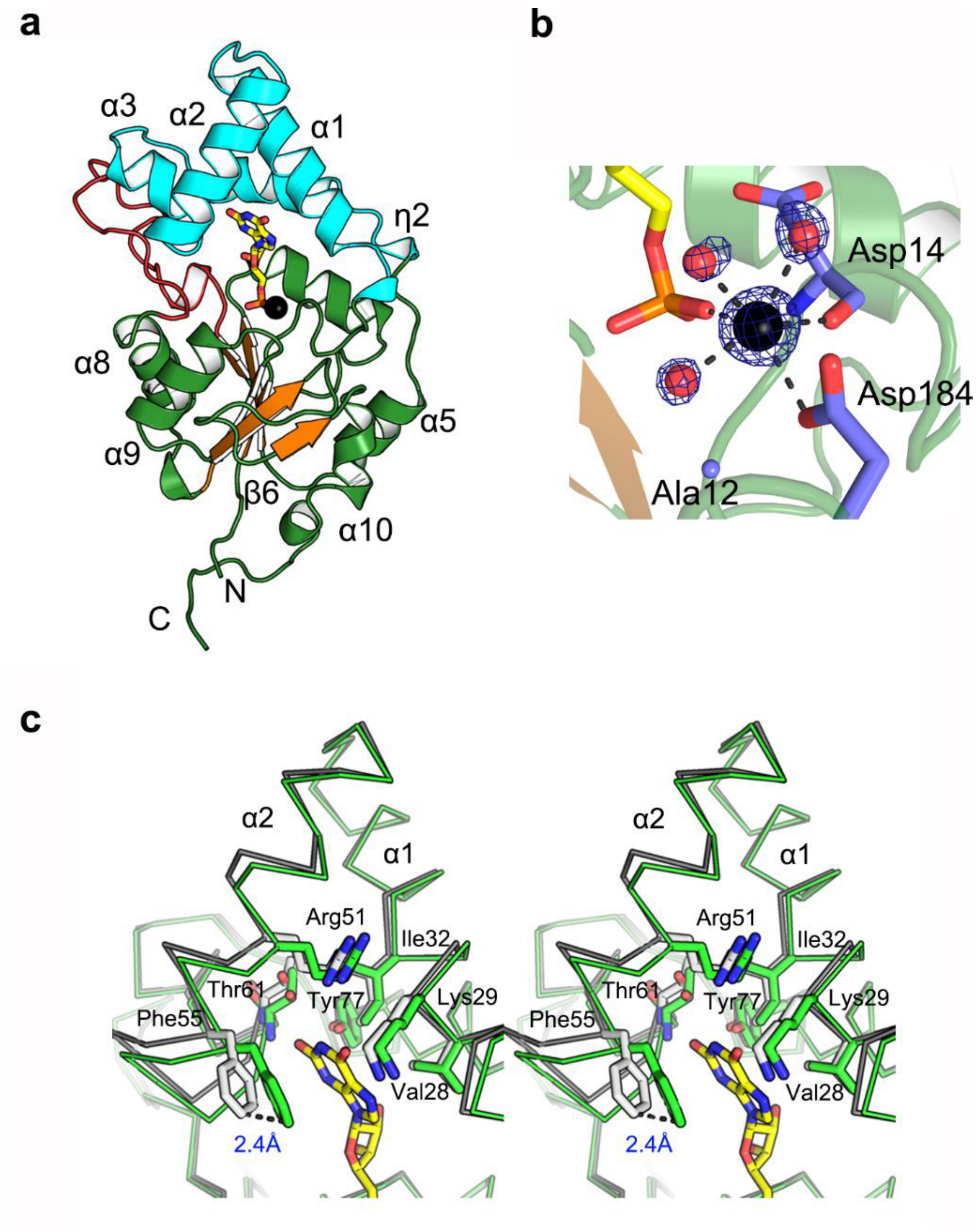

Supplementary Fig. 12. Structure of XMPP(D12A) in complex with XMP. **a** An overall structure of the XMPP(D12A)-XMP complex with a ball-and-stick presentation for XMP. Labels and color codes are identical to those in Fig. 4a. **b** Mg^{2+}-binding site in the XMPP(D12A)-XMP complex. Due to the mutation of D12A, the coordination of the metal ion (black sphere) is not in a square bi-pyramidal shell. Two water molecules above the metal ion are overlaid with a *2Fo-Fc* electron density contoured at 2.0 σ, with the other water molecules across Asp14 at 1.5 σ and the metal ion at 3.0 σ, respectively. **c** Comparison of the xanthine-binding environment of XMP for the unliganded XMPP (gray) and the complex (green). The two structures were superimposed using the central core domain, resulting in a root-mean square deviation (RMSD) of 0.66 Å for 145 Cα atoms. Note, there are no significant conformational changes in the binding environment, and the largest displacement of approximately 2.4 Å is observed for the CZ of Phe55. This region contains Phe55 and Thr61 that interact with the xanthine moiety (Fig. 4d). In the presence of XMP it shifts its position toward XMP without significantly perturbing any side chain conformations. Further analysis indicated that XMP is almost buried in the complex.

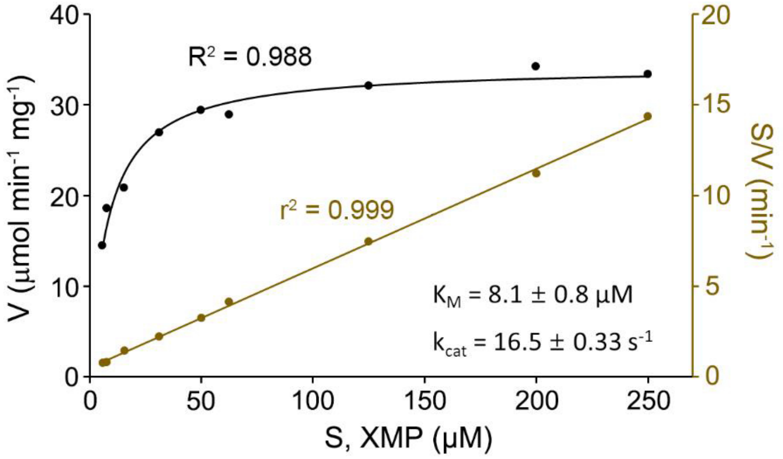

Supplementary Fig. 13. Steady-state kinetic analysis of C-terminal His-tagged full-length XMPP. The enzyme was expressed in *E. coli* and the enzyme assay performed at 30°C. Kinetic constants were determined by carrying out reactions containing 18 nM wild-type XMPP with 0 to 250 μM XMP.

Supplementary Table 1. Species names, protein abbreviations and corresponding locus identifiers (in Phytozome V12.1) for proteins used in the phylogenetic analysis

species	abbreviation	locus identifier	remark
Ananas comosus	Ana_com_1	Aco018447.1	Manually corrected according to consensus. Intron with GC acceptor site postulated.
	Ana_com_2a	Aco017201.1	
	Ana_com_2b	Aco013639.1	
	Ana_com_3A	Aco016820.1	
	Ana_com_3B	Aco001439.1	
Aquilegia coerulea	Aqu_coe_1	Aqcoe5G056900.1	
	Aqu_coe_2	Aqcoe2G238600.2	
	Aqu_coe_3	Aqcoe1G108100.2	
	Aqu_coe_3/4	Aqcoe2G223000.1	
Arabidopsis thaliana	Ara_tha_1	At2g32150.1	
	Ara_tha_2	At3g62040.1	
	Ara_tha_3	At5g02230.1	
	Ara_tha_4A	At5g59480.1	
	Ara_tha_4B	At5g59490.1	
Brachypodium distachyon	Bra_dis_1	Bradi1g11620.1	
	Bra_dis_2A	Bradi1g20280.2	
	Bra_dis_2B	Bradi1g02690.1	
	Bra_dis_3A	Bradi1g66710.1	
	Bra_dis_3B	Bradi2g62150.1	
Capsella rubella	Cap_rub_1	Carubv10025064m	
	Cap_rub_2	Carubv10019512m	Sequence manually corrected. Second intron has unusual GA-AG splice junctions and is not correctly annotated
	Cap_rub_3a	Carubv10001663m	
	Cap_rub_3b	Carubv10023829m	
	Cap_rub_4A	Carubv10026922m	
	Cap_rub_4Ba	Carubv10027751m	
	Cap_rub_4Bb	Carubv10028046m	
Carica papaya	Car_pap_1	evm.model.supercontig_58.124	
	Car_pap_2	evm.model.supercontig_57.45	

	Car_pap_3	evm.model.supercontig_169.20	
	Car_pap_4A	evm.model.supercontig_81.119	
	Car_pap_4B	evm.model.supercontig_81.118	
Citrus sinensis	Cit_sin_1	orange1.1g025190m	
	Cit_sin_2	orange1.1g024578m	
	Cit_sin_3	orange1.1g022360m	
	Cit_sin_4A	orange1.1g036723m	
	Cit_sin_4B	orange1.1g035566m	Genomic sequence has a gap. Translated mRNA sequence (accession XM_025102177.1) was used instead.
Medicago truncatula[1]	Med_tru_1	Medtr1g104610.1	
	Med_tru_2	Medtr1g085570.1	
	Med_tru_3a	Medtr4g021800.1	
	Med_tru_3b	Medtr7g062150.1	
	Med_tru_4a	Medtr2g097520.1	
	Med_tru_4b	Medtr4g064380.1	
Mimulus guttatus	Mim_gut_1	Migut.G00328.1	
	Mim_gut_2	Migut.L01000.1	
	Mim_gut_3a	Migut.L01953.1	
	Mim_gut_3b	Migut.J00320.1	
Oryza sativa	Ory_sat_1	LOC_Os03g49440.1	
	Ory_sat_2A	LOC_Os07g44060.1	
	Ory_sat_2B	LOC_Os03g61829.1	
	Ory_sat_3A	LOC_Os03g16670.1	
	Ory_sat_3B	LOC_Os01g74152.1	
Physcomitrium patens	Phy_pat_1	Pp3c27_8130V3.2	
Selaginella moellendorfii	Sel_moe_1	73861	Manually corrected according to consensus.
Setaria italica[1]	Set_ita_1	Seita.9G127000.1	
	Set_ita_2Aa	Seita.2G403800.1	
	Set_ita_2Ab	Seita.2G403900.1	
	Set_ita_2B	Seita.9G022100.1	
	Set_ita_3A	Seita.9G453700.1	
	Set_ita_3B	Seita.5G468600.1	
Solanum tuberosum	Sol_tub_1	PGSC0003DMT400081430	

	Sol_tub_2	PGSC0003DMT400002201	
	Sol_tub_3	PGSC0003DMT400022761	
	Sol_tub_4	PGSC0003DMT400078186	
Sorghum bicolor	Sor_bic_1	Sobic.001G125000.1	
	Sor_bic_2A	Sobic.002G388400.1	
	Sor_bic_2B	Sobic.001G027300.1	
	Sor_bic_3A	Sobic.001G422100.1	
	Sor_bic_3B	Sobic.003G444200.1	
Spagnum phallax	Spa_pha_1	Sphfalx0274s0003.1	
Vitis vinifera	Vit_vin_1	GSVIVT01017946001	
	Vit_vin_2	GSVIVT01028227001	Manually corrected according to consensus.
	Vit_vin_3	GSVIVT01034151001	Manually corrected according to consensus.
	Vit_vin_4	GSVIVT01024928001	
Zostera marina	Zos_mar1	Zosma185g00220.1	
	Zos_mar2	Zosma4g00550.1	
	Zos_mar3	Zosma241g00050.1	

[1] Sequences with strong deviations from consensus were not considered in the analysis: Medtr4g021825.1; Seita.2G403700.1.

Supplementary Table 2. Data collection and refinement statistics

Data set<br><br>PDB ID	XMPP<br><br>7EF6	XMPP(D12A) complexed with XMP<br>7EF7
**Data collection**		
Wavelength (Å)	0.97933	0.97933
Resolution (Å)	50.0 – 1.34 (1.39-1.34)[a]	50.0 – 1.50 (1.53-1.50)[a]
Unique reflections	51,330 (4,973)	37,816 (1,715)
Multiplicity	5.4 (4.9)	3.5 (2.9)
Completeness (%)	98.9 (95.4)	98.3 (91.4)
Mean I/sigma(I)	31.1 (3.0)	15.2 (1.0)
Wilson *B*-factors ($Å^2$)	16.4	20.5
R-merge	0.057 (0.391)	0.086 (0.875)
$CC_{1/2}$[b]	0.993 (0.969)	0.989 (0.622)
**Space group**	$P2_1$	$P2_1$
Unit cell *a, b, c* (Å)	37.1, 55.3, 57.2	37.7, 56.8, 57,0
α , β , γ (º)	90, 93.9, 90	90, 92.9, 90
**Refinement**		
R-work[c]	0.190	0.213
R-free[d]	0.226	0.240
No. of atoms		
Macromolecules	1828	1834
Ligands	1	25
Water	192	80
RMS (bonds) (Å)	0.012	0.009
RMS (angles) (º)	1.42	1.17
Ramachandran favored (%)	99.6	98.3
Ramachandran outliers (%)	0.0	0.0
Average *B*-factors ($Å^2$)		
Macromolecules	22.6	50.6
Ligands	16.3	43.2
Water	30.7	53.3

[a]Numbers in parentheses refer to data in the highest resolution shell.
[b]The $CC_{1/2}$ is the Pearson correlation coefficient (CC) calculated from each subset containing a random half of the measurements of unique reflection
[c]$R_{work} = \Sigma\,||F_{obs}|-|F_{cal}||\,/\,\Sigma|F_{obs}|$
[d]R_{free} is the same as R_{obs} for a selected subset (5%) of the reflections that was not included in prior refinement calculations.

Supplementary Table 3. Primer list and synthetic XMPP sequence

Primer	Sequence
N188	TCCATGGATTTCTCTCCGATCAACTGC
N189	ACCCGGGTCACGCACCGACGGCTGC
N359	TATCGATAAAATGGATTTCTCTCCGATCAACTGC
N360	TCCCGGGCGCACCGACGGCTGCTATT
1033	GTGAACGATTCCTGGACCTGCCTC
1034	GAGAGGTTACATGTTCACCACAAC
1709	TCGAGCTCAAAATGGGTTCTGCATGGTCTCATCCTCAATTTGAAAAAGGCGCCACCATGG
1710	AATTCCATGGTGGCGCCTTTTTCAAATTGAGGATGAGACCATGCAGAACCCATTTTGAGC
P243	TCCCGGGATGGTGAGCAAGGGCGAGGAGACCAC
P244	TGAGCTCTCACTTGTACAGCTCGTCCATGCCGTCGG
P1094	TGGCGCGCCTGCTGGTTAGATAGATTCATGGC
P1097	GCGCCGGTTCCGGTGGGGGCTCCGGGGGAAGTGCATGGTCCCATCCGCAGTTCGAGAAAGGTGGTTC
P1098	CATGGAACCACCTTTCTCGAACTGCGGATGGGACCATGCACTTCCCCCGGAGCCCCCACCGGAACCG
P1103	ACTCGAGTACAAGAGAACAAGTAAGTAAGAAAATATA
X0001	GGAGATATACATATGGATTTCAGCCCGATCACAA
X0002	GTGGTGGTGCTCGAGGCTTTTGCTACGACGGATA

Primers for the generation of point mutations

Primer	Name indicating mutation site	Sequence (mutation site in bold)
X0121	D12A_F	GCCCGATCAACTGCCTGATCTTT**GCG**CTGGATGACA
X0122	D12A_R	TGTCATCCAG**CGC**AAAGATCAGGCAGTTGATCGGGC
X0141	D14A_F	CTTTGACCTG**GCG**GACACCCTGTATCCGCTGAAGAC
X0142	D14A_R	GTCTTCAGCGGATACAGGGTGTC**CGC**CAGGTCAAAG
X0281	V28A_F	GTATCGCGCCGGCC**GCG**AAAAAAAACATCGATGAC
X0282	V28A_R	GTCATCGATGTTTTTTTT**CGC**GGCCGGCGCGATAC
X0291	K29A_F	AAGACGGGTATCGCGCCGGCCGTG**GCA**AAAAACAT
X0292	K29A_R	ATGTTTTT**TGC**CACGGCCGGCGCGATACCCGTCTT
X0295	K29R_F	CCGGC**CGT**GCGTAAAAACATCGATGACTTCCTGG
X0266	K29R_R	CCAGGAAGTCATCGATGTTTTTACGC**ACG**GCCGG
X0321	I32A_F	CCGGCCGTGAAAAAAAAC**GCG**GATGACTTCCTGG
X0322	I32A_R	CCAGGAAGTCATC**CGC**GTTTTTTTTCACGGCCGG
X0511	R51A_F	CTGAATCCAAAGCGTCCAGCCTG**GCG**GTAGAACTC
X0512	R51A_R	GAGTTCTAC**CGC**CAGGCTGGACGCTTTGGATTCAG
X0551	F55A_F	AGCCTGCGTGTAGAACTC**GCG**AAAACGTACGGGT
X0552	F55A_R	ACCCGTACGTTTT**CGC**GAGTTCTACACGCAGGCT
X0611	T61A_F	AGAACTCTTCAAAACGTACGGGTCT**GCG**CTGGCA
X0612	T61A_R	TGCCAG**CGC**AGACCCGTACGTTTTGAAGAGTTCT
X0615	T61V_F	AGAACTCTTCAAAACGTACGGGTCT**GTG**CTGGCA
X0616	T61V_R	TGCCAG**CAC**AGACCCGTACGTTTTGAAGAGTTCT
X0771	Y77F_F	TCCTGATGAA**TTT**CATTCTTTCGTTCATGGTCGTC
X0772	Y77F_R	GACGACCATGAACGAAAGAATG**AAA**TTCATCAGGA
X0781	H78A_F	GCCACGACGTTCATCCTGATGAATAC**GCG**TCTTT
X0782	H78A_R	AAAGA**CGC**GTATTCATCAGGATGAACGTCGTGGC
X0821	H82A_F	CCTGATGAATACCATTCTTTCGTT**GCG**GGTCGTCT
X0822	H82A_R	AGACGACC**CGC**AACGAAAGAATGGTATTCATCAGG
X1111	T111A_F	ATCAAACAGCGCAAAATTATCTTT**GCG**AATTCTGA
X1112	T111A_R	TCAGAATT**CGC**AAAGATAATTTTGCGCTGTTTGAT
X1121	N112A_F	TATCTTTACG**GCG**TCTGATAAAAACCATGCCGTCAA
X1122	N112A_R	TTGACGGCATGGTTTTTATCAGA**CGC**CGTAAAGATA
X1591	K159A_F	GGTTGTTCTG**GCG**CCGAGTCTGACCGCCATGGATA
X1592	K159A_R	TATCCATGGCGGTCAGACTCGG**CGC**CAGAACAACC
X1831	D183A_F	GTGTTCCTG**GCG**GATAACATTCACAACATCACCGC
X1832	D183A_R	GCGGTGATGTTGTGAATGTTATC**CGC**CAGGAACAC
X1841	D184A_F	GTTCCTGGAT**GCG**AACATTCACAACATCACCGCCG
X1842	D184A_R	CGGCGGTGATGTTGTGAATGTT**CGC**ATCCAGGAAC
X1881	N188A_F	AACATTCAC**GCG**ATCACCGCCGGTAAATCTGTAGGC
X1882	N188A_R	GCCTACAGATTTACCGGCGGTGAT**CGC**GTGAATGTT

Sequence of the synthetic *XMPP* gene for expression in *E. coli*

```
ATGGATTTCAGCCCGATCAACTGCCTGATCTTTGACCTGGATGACACCCTGTATCCGCTG
AAGACGGGTATCGCGCCGGCCGTGAAAAAAAACATCGATGACTTCCTGGTAGAGAAATTC
GGTTTCTCTGAATCCAAAGCGTCCAGCCTGCGTGTAGAACTCTTCAAAACGTACGGGTCT
ACCCTGGCAGGCCTGCGCGCACTGGGCCACGACGTTCATCCTGATGAATACCATTCTTTC
GTTCATGGTCGTCTGCCGTACGGTTCCATCGAACCTAACAACAAACTGCGTAACCTGCTG
AATAAAATCAAACAGCGCAAAATTATCTTTACGAATTCTGATAAAAACCATGCCGTCAAA
GTTCTGAAGAAGCTGGGCCTGGAGGACTGTTTTGAGGAAATGATTTGTTTCGAAACCATG
AACCCGAACCTGTTCGGTAGCACTACCCGTCCGGACGAATACCCGGTTGTTCTGAAACCG
AGTCTGACCGCCATGGATATCTGTATTCGCGTAGCAAATGTTGATCCGCGTCGTACCGTG
TTCCTGGATGATAACATTCACAACATCACCGCCGGTAAATCTGTAGGCCTGCGTACTATC
CTGGTGGGTCGCGCGGAAAAAACTAAAGATGCTGACTACGCAGTTGAAACCGTGACCGAA
ATCGCGACCGCAGTTCCGGAAATCTGGGCGACTGCGACCGCTACCGGCGGTTTCGACGTG
GGTGGAGAACGTATCCGTCGTAGCAAAAGCGAGCTGGAGGGTATGGCTTCTATTGCAGCT
GTTGGCGCGTGA
```

Supplementary Table 4. MS parameter

MS source parameter	Positive ion mode	Negative ion mode
**Ion source**	AJS ESI	AJS ESI
**Gas temperature**	300 °C	350 °C
**Gas flow**	12 L min^{-1}	12 L min^{-1}
**Nebulizer**	30 psi	40 psi
**Sheath gas heater**	300 °C	350 °C
**Sheath gas flow**	11 L min^{-1}	12 L min^{-1}
**Capillary**	4,000 V	2,500 V

Analyte	Ion mode	Retention time (min)	Precursor Ion (m/z)	Product ion[1] (m/z)	Frag-mentor V)	Collision energy (V)	Qualifier ratio
**allantoate**	pos.	0.80	177.05	117.0	60	25	
				74.0	60	25	21.9 – 32.6
**urate**	neg.	1.05	167.03	124.0	91	14	
				69.2	91	20	11.1 – 16.5
**xanthosine**	pos.	1.50	285.08	153.0	60	5	
				135.9	60	35	15.6 – 23.3
**guanine**	pos.	2.20	152.05	135.0	118	18	
				110.0	118	22	43.9 – 65.8
**guanosine**	pos.	5.00	284.09	152.0	90	10	
				135.0	90	45	26.0 – 39.5

[1] the first listed product ion was used for quantification.
