# Supplementary material for: Initiation of cytosolic plant purine nucleotide catabolism involves a monospecific xanthosine monophosphate phosphatase
Source: Nat Commun. 2021 Nov 25;12:6846. doi: 10.1038/s41467-021-27152-4 (PMC8616923; doi:10.1038/s41467-021-27152-4)
Supplement: Supplementary file 3 — Reporting Summary [file 41467_2021_27152_MOESM3_ESM.pdf]

nature research

Corresponding author(s): Claus-Peter Witte and Sangkee Rhee

Last updated by author(s): Oct 25, 2021

Reporting Summary

Nature Research wishes to improve the reproducibility of the work that we publish. This form provides structure for consistency and transparency in reporting. For further information on Nature Research policies, see our Editorial Policies and the Editorial Policy Checklist.

Statistics

For all statistical analyses, confirm that the following items are present in the figure legend, table legend, main text, or Methods section.

n/a	Confirmed	
☐	☒	The exact sample size (*n*) for each experimental group/condition, given as a discrete number and unit of measurement
☐	☒	A statement on whether measurements were taken from distinct samples or whether the same sample was measured repeatedly
☐	☒	The statistical test(s) used AND whether they are one- or two-sided<br>*Only common tests should be described solely by name; describe more complex techniques in the Methods section.*
☒	☐	A description of all covariates tested
☐	☒	A description of any assumptions or corrections, such as tests of normality and adjustment for multiple comparisons
☐	☒	A full description of the statistical parameters including central tendency (e.g. means) or other basic estimates (e.g. regression coefficient) AND variation (e.g. standard deviation) or associated estimates of uncertainty (e.g. confidence intervals)
☐	☒	For null hypothesis testing, the test statistic (e.g. *F*, *t*, *r*) with confidence intervals, effect sizes, degrees of freedom and *P* value noted<br>*Give P values as exact values whenever suitable.*
☒	☐	For Bayesian analysis, information on the choice of priors and Markov chain Monte Carlo settings
☒	☐	For hierarchical and complex designs, identification of the appropriate level for tests and full reporting of outcomes
☒	☐	Estimates of effect sizes (e.g. Cohen's *d*, Pearson's *r*), indicating how they were calculated

Our web collection on statistics for biologists contains articles on many of the points above.

Software and code

Policy information about availability of computer code

Data collection	Leica Application Suite X (Ver. 3.7.2.22383), MassHunter Workstation Software LC/MS Data Acquisition for 6400 Series Triple Quadrupole (Ver. B.07.00), UV Probe (Ver. 2.51), Spectra Manager (Ver. 2.10.01)
Data analysis	Excel 2016, Muscle (at the website of the European Bioinformatics Institute), MEGA X, GraphPad Prism 4, R software (Ver. 1.2.5042), Phyre2, XtalPred, HKL2000 software, PHENIX software including Ensembler and Sculptor utilities, COOT, SigmaPlot (Ver. 10.0), MassHunter Workstation Software Quantitative Analysis (Ver. B.09.00)

For manuscripts utilizing custom algorithms or software that are central to the research but not yet described in published literature, software must be made available to editors and reviewers. We strongly encourage code deposition in a community repository (e.g. GitHub). See the Nature Research guidelines for submitting code & software for further information.

Data

Policy information about availability of data

All manuscripts must include a data availability statement. This statement should provide the following information, where applicable:

- Accession codes, unique identifiers, or web links for publicly available datasets
- A list of figures that have associated raw data
- A description of any restrictions on data availability

The T-DNA mutants xmpp-1 (SALK067037) and xmpp-2 (SALK131244), nsh1 (SALK083120) and gsda (GK432D08) were used.
Sequence data from this article can be found with the following locus identifiers: XMPP, At2g32150; NSH1, At2g36310; GSDA, At5g28050, Actin2 At3g18780.
Atomic coordinates and structure factors of XMPP with and without XMP bound have been deposited in the Protein Data Bank (PDB) under accession codes 7EF7 and 7EF6, respectively.
Figures with associated raw data are:

Figures 1, 2, 3, 4
Supplementary Figures 2, 3, 4, 5, 6, 7, 8, 9, 13

Field-specific reporting

Please select the one below that is the best fit for your research. If you are not sure, read the appropriate sections before making your selection.

[x] Life sciences [ ] Behavioural & social sciences [ ] Ecological, evolutionary & environmental sciences

For a reference copy of the document with all sections, see nature.com/documents/nr-reporting-summary-flat.pdf

Life sciences study design

All studies must disclose on these points even when the disclosure is negative.

Sample size

For metabolite analyses, excluding nucleotide analysis, samples sizes of n=6 were chosen on the basis of preliminary experiments. As the preparations of nucleotide analysis were laborious, samples sizes of n=5 and n=3 were chosen for nucleotide analysis in seedlings and seeds, respectively. Due to small mean differences in the metabolite analysis of nsh1 gsda and xmpp nsh1 gsda seeds in preliminary experiments, corresponding sample sizes were determined by power analysis performed with R software using preliminary data.

Data exclusions

In the metabolite analysis measurements failing the pre-established quality criteria for retention time, qualifier to quantifier ratio or with a signal to noise ratio below 10 were called "not detected" (see Supplementary Table 4). If measurements failed due to technical reasons, they were called "not available" in the Extended Data Table 1.

Replication

All metabolite data, excluding nucleotide measurements, were reproduced at least once. In vitro enzyme activities were reproduced once. Subcellular localization was reproduced once. Enzyme activities were measured at least three times using the same enzyme preparation.

Randomization

Plants used for metabolite analysis were grown randomized.

Blinding

After plant harvest, samples were analysed in randomized and blind fashion.

Reporting for specific materials, systems and methods

We require information from authors about some types of materials, experimental systems and methods used in many studies. Here, indicate whether each material, system or method listed is relevant to your study. If you are not sure if a list item applies to your research, read the appropriate section before selecting a response.

Materials & experimental systems

n/a	Involved in the study	
[ ]	[x]	Antibodies
[x]	[ ]	Eukaryotic cell lines
[x]	[ ]	Palaeontology and archaeology
[x]	[ ]	Animals and other organisms
[x]	[ ]	Human research participants
[x]	[ ]	Clinical data
[x]	[ ]	Dual use research of concern

Methods

n/a	Involved in the study	
[x]	[ ]	ChIP-seq
[x]	[ ]	Flow cytometry
[x]	[ ]	MRI-based neuroimaging

Antibodies

Antibodies used

Mouse monoclonal anti-GFP antibody (11814460001, clones 7.1 and 13.1, Roche; https://antibodyregistry.org/search.php?q=AB_390913), custom-made rabbit polyclonal anti-XMPP antibody (ImmunoGlobe), anti-mouse IgG alkaline phosphatase conjugate antibody (A3562, Sigma-Aldrich, https://www.sigmaaldrich.com/certificates/COFA/A3/A3562/A3562-BULK_________SLCD1906__.pdf), anti-rabbit IgG horseradish peroxidase conjugate antibody (RABHRP1, Sigma-Aldrich).

Validation

Mouse monoclonal anti-GFP antibody and anti-mouse IgG alkaline phosphatase conjugate antibody were validated by long-lasting experience in many experiments of our laboratory. Custom-made anti-XMPP antibody was confirmed to detect the presence of XMPP protein in wild type and complementation lines and the absence of XMPP protein in mutant lines (Supplementary Fig. 3c). This experiment also validated the functionality of the RABHRP1 secondary antibody.
